# Dynamic wavelet correlation analysis for multivariate climate time series

**DOI:** 10.1038/s41598-020-77767-8

**Published:** 2020-12-04

**Authors:** Josué M. Polanco-Martínez, Javier Fernández-Macho, Martín Medina-Elizalde

**Affiliations:** 1 Basque Centre for Climate Change (BC3), 48940 Leioa, Spain; 2 Dept. of Quantitative Methods, University of the Basque Country, 48015 Bilbao, Spain; 3 Dept. of Geosciences, University of Massachusetts, Amherst, MA USA

**Keywords:** Climate sciences, Atmospheric science, Climate change, Palaeoceanography, Palaeoclimate

## Abstract

The wavelet local multiple correlation (WLMC) is introduced for the first time in the study of climate dynamics inferred from multivariate climate time series. To exemplify the use of WLMC with real climate data, we analyse Last Millennium (LM) relationships among several large-scale reconstructed climate variables characterizing North Atlantic: i.e. sea surface temperatures (SST) from the tropical cyclone main developmental region (MDR), the El Niño-Southern Oscillation (ENSO), the North Atlantic Multidecadal Oscillation (AMO), and tropical cyclone counts (TC). We examine the former three large-scale variables because they are known to influence North Atlantic tropical cyclone activity and because their underlying drivers are still under investigation. WLMC results obtained for these multivariate climate time series suggest that: (1) MDRSST and AMO show the highest correlation with each other and with respect to the TC record over the last millennium, and: (2) MDRSST is the dominant climate variable that explains TC temporal variability. WLMC results confirm that this method is able to capture the most fundamental information contained in multivariate climate time series and is suitable to investigate correlation among climate time series in a multivariate context.

## Introduction

The climate system is highly dynamic and involves interactions among five main Earth system components or subsystems: the atmosphere, the hydrosphere, the cryosphere, the lithosphere and the biosphere^1,2^. Processes taking place within these components and during their interactions occur at different spatial and temporal scales, from molecular to planetary levels and from seconds to millions of years^1,3^. These Earth system components interact through mass, energy and momentum exchanges creating feedback loops and chains (the cascade effect) despite the climate system itself being a closed system (the climate system is considered as a closed system, i.e. this system does not exchange mass outside of the system, although there is still exchange of energy^3,4^). The climate system is also under the influence of external forcings, such as aerosol emissions from volcanic eruptions, changes in Earth’s orbital parameters (astronomical forcing), fluctuations of solar radiation and increases in the atmospheric composition of greenhouse gases due to human activities^1,5^. Each subsystem affects the response of another, ultimately determining a climate state. Interactions among the different parts of the climate system generate disproportionate relations between inputs and outputs, and therefore originating a complex, non-linear and non-stationary dynamic system^4,6^.

The complexity, nonlinearity and nonstationarity of the climate system is reflected by the resulting essential climate variables—ECV (variables that play a critical role in characterizing the Earth’s climate). Such climate variables are represented as time series, for example, of atmospheric or ocean surface temperature, ocean salinity, precipitation, etc.^4,7,8^. In addition to the nonlinearity and nonstationarity nature of climate dynamics, time series of climate variables used to characterize it also have other properties irrespective of their source (i.e. archives of climate information come mainly from direct instrumental measurements or indirect evidence, from climate model simulations, and paleoclimate reconstructions). For example, typically, climate time series show strong autocorrelation (memory or persistence), are generally short, noisy, contain uncertainty, may be unevenly spaced, may contain periodic, quasi-periodic or transient signals, and are a composition of numerous packages of information in time-scales (i.e. multiscale phenomena)^3,8–12^.

A useful approach to extract information from climate time series is the application of diverse kinds of statistical methods, particularly time series analysis and signal processing techniques^3,8,13,14^. Statistical data analysis are traditionally used in climate research in support of scientific affirmations: in order to estimate and assign confidence intervals around observations and predictions, to detect non-negligible noise level in climate variables and to identify fundamental relationships among them^3,10^. Regarding the latter, there are a great number of techniques to study the relationship between two climate time series (bivariate analysis): from the classical Pearson’s and Spearman’s correlation or the cross-correlation function (CCF)^3,10^, binned^8,15^ and synchrony^8^ correlation to most sophisticated techniques such as kernel methods^16,17^, cross-recurrence plots^18,19^, and wavelet correlation^20,21^ and wavelet coherence^22–25^. In contrast, large number of techniques are not available to study the relationship among multiple variables.

Among the correlation techniques, the wavelet correlation either via the discrete wavelet transform (DWT) or especially via the continuous wavelet transform (CWT), is highly used by climatologists and paleoclimatologist and by other environmental research communities^11,20,22–28^. The wavelet correlation via the wavelet transform (WT) can be seen as an “improved version” of the combination of Fourier transform and partial and sliding correlations in different periods (known as windowed or short-time Fourier transform—WFT^29^) although methodologically are different. The WFT represents an inaccurate and inefficient method of time–frequency localization, since it imposes a scale or “response interval” into the analysis, whereas the WT is a method of time-frequency localization that is scale independent^22,30^. The wavelet (uni and bivariate) analysis is an adequate and versatile mathematical tool to tackle several characteristics of climate time series, such as nonstationarity, in search of (quasi) periodical or oscillatory signals, and to examine multiscale phenomena. Bivariate wavelet analysis also permits to study the relationship between two climate time series and is particularly appropriate for tracking dual change in forcing by exogenous variables^20,22,24,25,28,31^. However, the use of wavelet correlation has been limited since its inception to the bivariate case (some exceptions are^32^ and^33^), and the study of climate dynamics usually involves more than two variables interacting with each other simultaneously^1,3^. For this reason, a multivariate version of the wavelet correlation has been required.

In line with this interest, the aim of this paper is to introduce for the first time to the climate community, and to those of related fields, the application of wavelet local multiple correlation (WLMC)^34^ to analyze “dynamically” (i.e. through time) over different time-scales, multivariate climate time series. Particularly, to examine relationships among climate variables known to be physically related to each other. This method improves our ability to understand the underlying mechanisms driving climate change on different timescales. Climate variables, and the nature of the relationship among them, change over time and thus cannot be accurately understood using conventional statistical methods that do not take into account their time evolution. The best approach is therefore to use correlation methods and computational tools that address non-stationary relationships among multiple climate variables, such as the wavelet local multiple correlation (WLMC)^34^.

The WLMC measures a non-stationary time-evolving correlation structure at different scales within a multivariate set of data, and consists of one single set of multiscale correlations along time, each of them calculated as the square root of the regression coefficient of determination in that linear combination of locally weighted wavelet coefficients for which such coefficient of determination is a maximum^34,35^. The WLMC is a powerful statistical and computational tool that was originally developed to estimate correlation among multivariate, non-stationary, financial time series^34,35^. However, as we demonstrate in this study, the WLMC has a strong potential to be used with multivariate, stationary and non-stationary, climate and environmental time series as well. It is important to introduce this tool to the climate community since the economic and financial communities have poor connection with the climate community, despite some international networks and initiatives to bring them together (e.g. Climate Econometrics, https://www.climateeconometrics.org/). In this study we also improve graphical outputs of the WLMC and propose a didactic and useful way to visualize the “dominant” variable(s) that maximizes multiple correlation through time for a set of climate time series. This is the first time that this kind of graphical representation for the WLMC is presented (please look at Fig. 3 right).

This study exemplifies the use of WLMC by analyzing multivariate paleoclimate time series. Specifically, we examine relationships among various large-scale climate proxy reconstructions spanning the Last Millennium (LM) that are known to influence tropical Atlantic hurricane frequency and intensity. These variables come from^36,37^ and represent: (1) sea surface temperatures (SST) anomalies in the main developmental region (MDR) for tropical cyclones (MDRSST); (2) the El Niño-Southern Oscillation (ENSO) sea surface temperature anomalies (ENSOSSTs, or ENSO hereafter); (3) the Atlantic Multidecadal Oscillation (AMO) sea surface anomalies (AMOSSTs, or AMO hereafter); and (4) tropical cyclone counts (TCC, or TC hereafter) as a proxy of cyclone activity. We examine ENSO, AMO and MDR SSTs because they are known to influence North Atlantic tropical cyclone activity and their underlying processes are still under investigation. These climate variables have been invoked to explain climate dynamics inferred from paleoclimate records on previous studies; e.g. Ref.^26^.

## Results

### North Atlantic tropical cyclone activity over the past 1500 years

**Figure 1:**
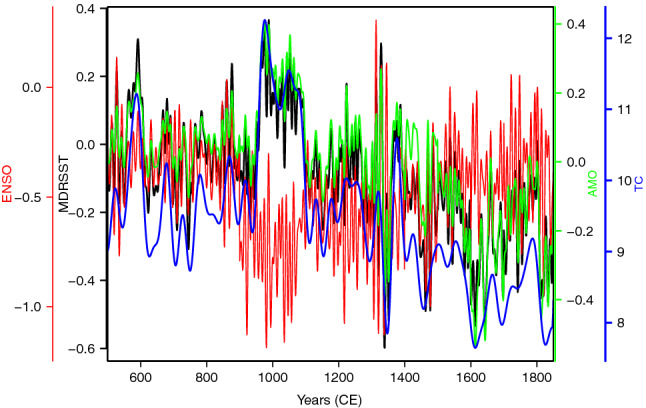
Sea surface temperature (SST) for the main development region (MDR) for Atlantic tropical cyclones^36^ (black); El Niño-Southern Oscillation (ENSO) SST (based on El Niño 3 region)^37^ (red); North Atlantic Atlantic Multidecadal Oscillation (AMO) SST averaged over the North Atlantic ocean^37^ (green); Long-term Atlantic tropical cyclone counts (TC)^36^ (blue). Climate variables are in anomalies ($^\circ$C), cover the time interval CE 500–1850, and the number of elements is 1350.

Figure 1 shows the climate time series examined in this study. Because MDRSST, AMO and TC have similar variability patterns through time, it is expected for them to have a relative high degree of correlation at “global” level, which can be corroborated by estimating the degree of correlation with each other. The correlation matrix obtained via Spearman’s rank correlation (Table 1) shows that the highest correlation ($r=0.910$) corresponds to the pair MDRSST–AMO, followed by the pairs AMO–TC ($r=0.843$) and MDRSST–TC ($r=0.774$). In contrast, ENSO shows an inverse relationship with AMO and TC (except relative to MDRSST which is direct), reflected by the negative correlation coefficients. This is confirmed by the pairwise comparison between ENSO and TC ($r=-0.296$) and between ENSO and AMO ($r=-0.132$). The negative correlation between ENSO and TC is expected because tropical cyclones can be suppressed when El Niño increases wind shear in the tropical Atlantic and warm upper tropospheric temperature anomalies^38–40^. On the other hand, the negative or inverse correlation between ENSO and AMO can be explained straightforwardly by the fact that during the negative AMO phase, both El Niño and La Niña events tend to be stronger than during a positive AMO phase^41^. Moreover, this inverse correlation between ENSO and AMO can be explained by the so-called atmospheric “bridge-thermocline feedback”^41,42^.

**Table 1:** Correlation matrix estimated through Spearman’s rank correlation.

	MDRSST	ENSO	AMO	TC
MDRSST	1.000	**0.155**	**0.910**	**0.774**
ENSO	**0.155**	1.000	**− 0.132**	**− 0.296**
AMO	**0.910**	**− 0.132**	1.000	**0.843**
TC	**0.774**	**− 0.296**	**0.843**	1.000

Statistically significant (95%) correlation coefficients are in bold.

### Wavelet local correlation for the bi-variate case for MDRSST, ENSO, and AMO

**Figure 2:**
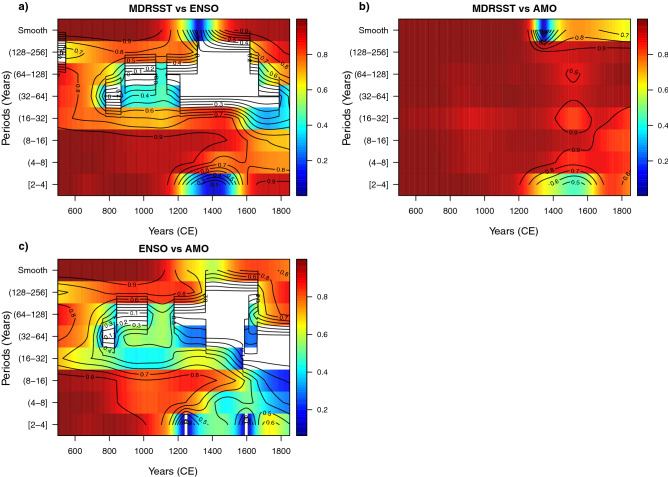
Wavelet local multiple correlation (bivariate case) for climate variables MDRSST, ENSO, and AMO. The time-period points in blank indicate that these points are not statistically significant (outside of the 95% confidence interval). WLMC parameters: M = 1350/8 (168) years, window = Gaussian, and wavelet filter (wf) = “la8”.

The wavelet local multiple correlation for the bivariate case (Fig. 2) is used as proof of concept for the multivariate case ($n > 2$), in order to determine the extent to which the climate variables by pairs are correlated with each other in the time and frequency (period) domains and thus avoid using the WLMC as a “black box”. The first evident result for this bivariate case is that MDRSST–AMO shows the highest WLMC among the three pairwise comparisons, in agreement with the result above using Spearman. However, one conspicuous feature of the dynamic correlation methods, such as the WLMC, is that they provide the evolution of correlation in the time and period domains. For example, the WLMC shows that the correlation of MDRSST–AMO is high (with coefficient values $> 0.80$) for practically all the periods considered and during almost the full time interval of the records (CE 500–1850), except for a small interval between CE 1400 and 1600, for the shortest scales (2–4 years). This result must be regarded with caution, however, because climate time series are decadally smoothed^36,37^. The second important result is that although the correlations of MDRSST–AMO is generally high, the strength of correlations tends to “oscillate” in time and nearly in all periods, indicating that the degree of correlation is not constant through time. This result has important implications for diagnosing the causal relationships among these variables and for understanding their underlying large-scale dynamics^36,43^. Lastly, the WLMC of ENSO–MDRSST and ENSO–AMO shows moderate degree of correlation that is well localized in the frequency/period domain. As expected, the highest correlation among the variables occurs at 4–8 and 8–16 year periods, where ENSO has its main spectral signature^36,40^. Furthermore, there is a “slowdown” in correlation at the end of the heat maps for both pairs (more pronounced for ENSO–AMO), and their corresponding time intervals are not exactly the same as for MDRSST–AMO.

### WLMC for the three-variate case for MDRSST, ENSO, and AMO

**Figure 3:**
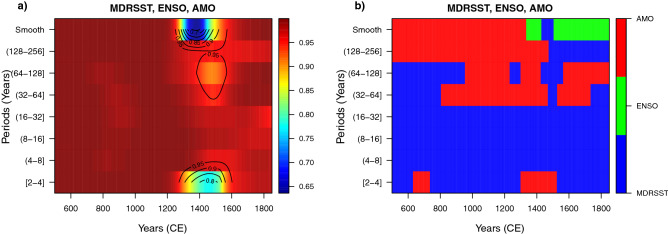
Wavelet local multiple correlation (tri-variate case) for climate variables MDRSST, ENSO, and AMO (left). Heat map that indicates the (“dominant”) variable(s) that maximizes the multiple correlation through time and scale (right) (blue, green, and red indicate MDRSST, ENSO, and AMO, respectively). The time-period points in blank indicate that these points are not statistically significant (outside of the 95% confidence interval). WLMC parameters: M = 1350/8 (168) years, window = Gaussian, wavelet filter (wf) = “la8”, and ymaxr = NULL.

Figure 3 shows the WLMC for the three-variate case. We do not define *a priori* specific climate variable that would maximize the multiple correlation for each wavelet scale (parameter ymaxr=NULL) but instead let the WLMC select one. The reason for this choice is that although these climate variables are expected to be correlated with each other, their causal relationship in time and frequency domains remains a matter of research. The three-variate WLMC shows a significant level of correlation with variable coefficients (from $\sim 0.8$ to $\sim 0.99$), from short to long periods of variability, and for practically the full length of the records. The three-variate WLMC result is quite similar to the WLCM for the pair MDRSST–AMO from the bivariate case (Fig. 2). This means that the WLMC method developed by^34^ is an “inclusive” multivariate correlation tool unlike the multiple wavelet coherence, that is “exclusive”^32,44,45^. For this reason, it is important to take into account, that the WLMC retains practically all the statistically significant correlations between dominant variables (e.g. MDRSST and AMO from this case study), but also considers the correlation among other variables (e.g. MDRSST–ENSO and ENSO–AMO). Importantly, the WLMC provides the “dominant” variable (the one that maximizes the multiple correlation and can be used to explain the other variables across each period and time interval) (Fig. 3 right). For example, for the triad MDRSST–ENSO–AMO, the MDRSST represents the dominant climate variable (followed to a lesser extent by AMO). This result is explained because SST fields are used to build MDRSST and also to estimate the climate indices of AMO and ENSO (see Supplementary Material in^37^).

### WLMC for the bi-variate case for MDRSST, ENSO, and AMO vs. TC

**Figure 4:**
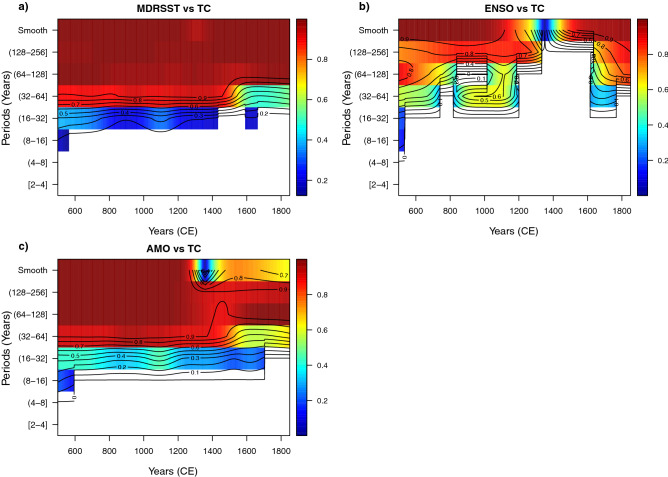
Wavelet local multiple correlation (bivariate case) for climate variables MDRSST, ENSO, AMO and TC. The time-period points in blank indicate that these points are not statistically significant (outside of the 95% confidence interval). WLMC parameters: M = 1350/8 (168) years, window = Gaussian, and wavelet filter (wf) = “la8”.

The WLMC for the bivariate case (Fig. 4), when the TC record is included shows three interesting results. The WLMC only shows statistically significant correlation coefficients with relatively high coefficients (approximately greater than 0.6) for medium (32–64 years) and long (128–256 years) periods. This result indicates that the WLMC tool works properly since the TC record is smoothed multidecadally ($> 40$ years) and therefore is not expected to reveal significant variability at periods lower than 40 years. The second important result is that apart from the high and statistically significant correlation for the pairs SSTMDR–TC and AMO–TC (please note that this result is in agreement with the correlation matrix presented previously in Table 1), the coefficient values tend to vary as a function of time, which is more evident at the periods 32–64 and 64–128 years for the pair SSMDR–TC and at the periods 32–64 and 128–256 years for the pair AMO–TC. The tool also shows an almost complete lack of statistically significant correlation between ENSO and TC, except for the long-term periods between 128 and 256 years. This lack of correlation reflects the smoothing of the TC record ($> 40$ years) relative to the main periods of ENSO variability (3–7 years).

### WLMC for the three- and four-variate cases for MDRSST, ENSO, and AMO vs. TC

**Figure 5:**
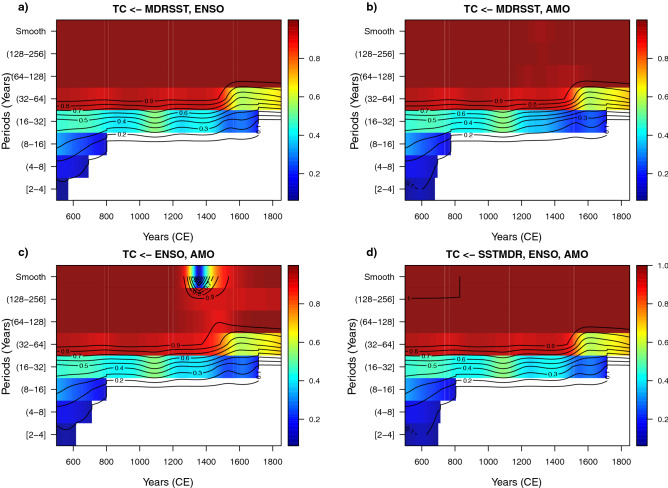
Wavelet local multiple correlation (tri- and tetra-variate cases) for climate variables MDRSST, ENSO, AMO and TC. The time-period points in blank indicate that these points are not statistically significant (outside of the 95% confidence interval). WLMC parameters: M = 1350/8 (168) years, window = Gaussian, wavelet filter (wf) = “la8”, and ymaxr = TC.

Figure 5 shows the three- and tetra-variate WLMC cases between the examined climate variables (MDRSST, ENSO and AMO) relative to the TC record. In these cases, the TC record is chosen to be the dependent variable (ymaxr=TC). The three- and tetra-variate cases confirm some of the previous results obtained for the bivariate case; e.g. statistically significant correlation from medium (16–32) to long (128–256) periods. An outstanding feature observed in these WLMC heat maps is that, despite the low or lack of correlation between ENSO and TC over several periods, the correlation between MDRSST and AMO relative to TC is preserved. This corroborates that the WLMC is an “inclusive” multivariate correlation tool, as previously pointed out. This means that, at least with these climate variables, the tetra-variate case does not provide “extra” information to be considered.

## Discussion

To summarize, this study introduces for the first time to the climate community and related fields the use of wavelet local multiple correlation (WLMC)^34^ to perform dynamic correlation analyses for multivariate climate time series. This method is suitable to compare time series that are linear, non-linear, non-stationary, containing (quasi)periodic events or transient signals, and that are a composition of numerous packages of information in time-scales ranging from days to millennia (i.e. multiscale phenomena).

This study exemplifies the use of WLMC to analyze a multivariate set of climate time series. Specifically, it examines relationships among various climate proxy reconstructions spanning the last millennium that are known to influence tropical Atlantic hurricane frequency and intensity. These variables come from^36,37^ and represent: sea surface temperatures anomalies in the main developmental region for tropical cyclones (MDRSST); the El Niño-Southern Oscillation sea surface temperature anomalies (ENSO); (3) the Atlantic multidecadal Oscillation (AMO) sea surface anomalies; and (4) the tropical cyclone counts (TC).

The WLMC results obtained for the multivariate paleoclimate time series confirm that MDRSST and AMO are the highest correlated variables among the climate data examined and with respect to TC. The MDRSST is found to be the dominant climate variable that can be used to explain the other variables examined. We would like to highlight that, the WLMC method developed by^34^, is an “inclusive” multivariate correlation tool. For this reason, it is important to take into account that the WLMC retains the statistically significant correlation between dominant variables (e.g. MDRSST and AMO), but also considers the correlation (statistically significant) among other variables (e.g. MDRSST and ENSO or ENSO and AMO). The WLMC is shown to be a suitable tool to investigate correlation among climate time series in a multivariate context.

## Methods

This section presents and discusses the methodological approach proposed and used in this study; the wavelet local multiple correlation (WLMC)^34^. We will follow^34,35,46,47^ to summarize the method. Readers interested in obtaining more detailed information can consult these references. The WLMC method is implemented by a freely available R computer package called *wavemulcor* with its corresponding documentation to perform the WLMC analysis^35,47^.

### Local multiple regression

The WLMC is based on the notion of multiple regression and in this paper we use the concept of wavelet local multiple regression introduced by^34,46^.

Let *X* be a multivariate time series of dimension *n* observed at times $t=1, \ldots, T$. According to^34^, for some $x_i \in X$ a local regression at a fixed $s\in [1,\ldots ,T]$ can be used to minimize the weighted sum of squared errors

1 $$\begin{aligned} S_s = \sum _{t} \theta (t-s)[f_s(X_{-i,t}) - x_{it})]^2 \end{aligned}$$

where $\theta (x)$ is a given moving average weight function that depends on the time lag between observations $X_t$ and $X_s$ and $f_s(X_{-i})$ is a local function of $\{X\!\!\setminus \!\!x_i\}$ around *s*. Letting *s* move along time, the corresponding local coefficients of determination are given by

2 $$\begin{aligned} R_s^2 = 1 - \frac{RwSS_s}{TwSS_s}, \;\;\; s=1,\ldots,T, \end{aligned}$$

where $RwSS_s$ and $TwSS_s$ are the residual and total weighted sum of squares respectively.

### Definition and estimation of the wavelet local multiple correlation

Let $W_{jt} = (w_{1jt},\ldots,w_{njt})$ be the wavelet coefficients for scale $\lambda _j$ (where $j=1,\ldots,J$, and *J* indicates the maximum level of the wavelet transform decomposition) obtained by applying the MODWT to each time series $x_i \in X$, where $i=1,\ldots,n$. Following to^34^, at each wavelet scale $\lambda _j$ the wavelet local multiple correlation coefficients ${\varphi }_{X,s}(\lambda _j)$ can be estimated as the square roots of the regression coefficients of determination for that linear combination of variables $w_{ij},$ $i=1,..,n$, where such coefficients of determination are maxima. That is, from Eq. (2)

3 $$\begin{aligned} \widetilde{\varphi }_{X,s}(\lambda _j) = \sqrt{R_{js}^2}, \;\;\; j=1,\ldots,J, \;\;\; s=1,\ldots,T. \end{aligned}$$

On the other hand, since the $R^2$ coefficient in the regression of a $z_i$ on the rest of variables in the system is equivalent to the square correlation between the observed and the fitted values $\widehat{z}_i$ obtained from such regression, according to^34^, it is possible to express the consistent sample estimator of the WLMC as

4 $$\begin{aligned}&{\widetilde{\varphi }}_{X,s}(\lambda _j) = \mathrm {Corr}\left( \theta (t-s)^{1/2}w_{ij}, {\theta }(t-s)^{1/2}\widehat{w}_{ij}\right) \nonumber \\&\quad s=1,\ldots,T, \end{aligned}$$

where ${w}_{ij}$ is chosen so that its local regression on the set of regressors $\{{w}_{kj}, k\ne i\}$ maximizes the corresponding coefficient of determination and $\widehat{w}_{ij}$ denotes the corresponding vector of fitted values.

### Weight (window) functions

The *wavemulcor* software^35,47^ used to estimate the WLMC includes six of the most commonly used weight functions (or windows) for averaging and smoothing: the uniform window, Bartlett’s triangular window, Cleveland’s tricube window, Wendland’s truncated power window, Epanechnikov’s parabolic window, and the Gaussian window. All six windows $\theta (x)$ satisfy $\int _{-\infty }^{\;\;\infty } \theta (x) dx = 1$ and have compact support in $\mid x \mid \le M$, where *M* is the length of the weight function $\theta (x)$, except the Gaussian window that takes values for $x \in (-\infty , \infty )$. However^34^, suggested that the uniform, Cleveland’s tricube and Epanechnikov’s parabolic windows are not recommended due to the presence of negative values in their corresponding spectral windows and, he also suggested to use the Bartlett’s triangular, Wendland’s truncated power, and Gaussian windows because these windows are most adequate for signal extraction and smoothing and their spectral windows are non-negative. By default, *wavemulcor*^35,47^ uses the Gaussian window for the following reasons: it is closest to the uniform weights (windows) in the time domain within a certain bandwidth, its Fourier transform is also Gaussian, it has near compact support in the frequency domain, and its spectral window is always positive^34^.

### Estimation of statistical significance

One of the main advantages of wavelet correlation obtained through the discrete wavelet transform (DWT), or its improved versions such as the MODWT, as compared to the continuous wavelet transform (CWT) is that for the (MO)DWT, it is possible to construct an analytical confidence interval (CI), while for the CWT it is practically impossible to do so.

In particular, for the WLMC^34,46^, obtained the CI by means of the Fisher’s transform as follows.

Let ${{\widetilde{\varphi }}}_{X,s}(\lambda _j)$ be the sample wavelet local multiple correlation (WLMC) calculated from Eq. (4). Then, from [^34^, Theorem 1],

5 $$\begin{aligned} \widetilde{Z}_{j,s} \overset{a}{\sim } \mathscr {F}\!\mathscr {N}(Z_{j,s}, (T/2^j - 3)^{-1}) \end{aligned}$$

where $\widetilde{Z}_{j,s} = \mathrm {arctanh}({\widetilde{\varphi }}_{X,s}(\lambda _j))$, $Z_{j,s} = \mathrm {arctanh}(\varphi _{X,s}(\lambda _j))$ and $\mathscr {F}\!\mathscr {N}$ stands for the folded normal distribution. Thus, since ${\varphi }_{X,s}(\lambda _j)$ is the correlation between weighted observations from two Gaussian variates of which $T/2^j$ are serially uncorrelated asymptotically, applying the Fisher’s transformation to $\epsilon _j$ such that $\mathrm {abs}(\epsilon _j) = \widetilde{Z}_{j,s}$ in Eq. (5)^34^, obtained the CI for the WLMC as

6 $$\begin{aligned} CI_{1-\alpha }({\varphi }_{X,s}(\lambda _j)) = \mathrm {tanh}\Big [ \widetilde{Z}_{j,s} \; \pm \; \phi _{1-\alpha /2}^{-1}/\sqrt{T/2^j - 3}\Big ] \end{aligned}$$

where $\phi _p^{-1}$ is the 100p% point of the standard normal distribution.

### Computational and practical aspects

In practice, the WLMC^34^ method consist of the following steps (the R code used to generate all the figures in this paper are in the Supplementary Information (Material)):

1. Estimation of the MODWT via the R package *waveslim*^48^ for each of the time series $x_i$ in the multivariate set ($i=1,\ldots,n$, where *n* is the number of time series) and all scales $\lambda _j$ ($j=1,\ldots,J$, where *J* is the maximum level of the MODWT decomposition with $J\le \log _2(T)$ and *T* is the length of the time series. Note that although the theoretical maximum level of the MODWT decomposition is given by $\log _2(T)$, in practice *J* should be much smaller in order to avoid boundary effects since the number of feasible wavelet coefficients becomes critically small for higher levels). As a wavelet filter or function, we chose the Daubechies *LA*(8) (or “la8”) that is aleast asymmetric wavelet filter of length $L=8$^49^. We use *LA*(8) since^20^ proposed this filter to analyze climate time series and^34,49,50^ showed that the use of a relatively long wavelet filter (e.g. *LA*(8) or *LA*(4)) is adequate to analyse non-stationary time series but also correlation structures that are not stationary. We used both wavelet filters (*LA*(8) and *LA*(4)) in all the examples presented in this paper and the WLMC heat maps are quite similar. In addition to these wavelet filters we used other eight filters with different lengths: “Haar” (L=2), “d4” (L=4), “d6” (L=6), “fk8” (L=8), “bl14” (L=14), “mb16” (L=16), “la20” (L=20), and “fk22” (L=22) and the WLMC heat maps (results not shown, but can be obtained through the R code included in the Supplementary Information (Material) or upon request to the corresponding author) are considerably similar to the corresponding one when the “la8” is used, except for “bl14”, “mb16”, and “fk22” and for the longest wavelet scales (periods), which can be explained mainly due to the “excessive” length of these wavelet filters. As a good practice, it is highly recommendable to try several wavelet filters (the MODWT from the R package *waveslim*^48^ includes 21 wavelet filters that can be used in the estimation of the WLMC) with different lengths (*L*), from short, medium to long, to study the sensitivity of the wavelet functions in order to corroborate the stability of the WLMC.
2. Application of the rolling time window or weight function $\theta (x)$ to the MODWT components. We used the default Gaussian window due to the aforementioned features. However, the other two recommendable options, Bartlett’s triangular or Wendland’s truncated power, provide similar results. Following to^35,47^, the recommended length of the weight function or rolling window is given by *T*/8, where *T* is the number of elements of time series. For the example presented in this paper we also choose a window length of *T*/8 (or 168 years since $T = 1350$) years as a compromise. A shorter window length would not have had enough data points to study longer climate time-scales phenomena and also could introduce a high degree of variability in the time domain, and a longer window length would have isolated climate phenomena that take place at short time scales. However, it is highly recommendable to try different window lengths, from short, medium, to long sizes (see e.g.^51–53^). For instance, in the example presented in this paper, in addition to the window length of *T*/8 (168 years), we tried other four window lengths ($M =$ 42, 84, 337, and 675 years) and the WLMC heat maps for 84 and 337 years are quite similar (whereas the “extreme” M values of 42 and 675 years are not very different) to that of the corresponding one with a value of $M=168$ years (results not shown, but can be obtained through the R code included in the Supplementary Information (Material) or upon request to the corresponding author).
3. Estimation of local least-squares regression applied to the wavelet coefficients $W_{ij}$ as described in^34^. At each wavelet level the implementation of WLMC in *wavemulcor*^35,47^ automatically chooses the variable maximizing the multiple correlation. Alternatively, the user may provide information about the number of the variable (parameter “ymaxr”) whose correlation against a linear combination of the others is to be calculated^35,47^. This may be useful if a relationship between the variables under study is known a priori.
4. Estimation of the wavelet local multiple correlation coefficients $\widetilde{\varphi }_{X,s}(\lambda _j)$ applying the consistent estimator in Eq. (3) and estimation of the confidence interval (Eq. 6) to establish the statistical significance of $\widetilde{\varphi }_{X,s}(\lambda _j)$.

### Datasets

To illustrate the WLMC method, we analyse the dynamic relationship among three large-scale climate variables closely related to each other and to tropical Atlantic cyclone activity from a multivariate perspective. These climate variables (Fig. 1) are based on paleoclimate reconstructions and cover the interval CE 500–1850^36,37^. They represent: (1) a record of sea surface temperature (SST) anomalies ($^\circ$C) from the main developed region (MDR) for tropical cyclones^36^. The MDRSST record shows interannual to multidecadal scale variability and reflects the “favourability” of the local thermodynamic environment to tropical cyclones formation^36,54–56^. However, we are aware that there has been a considerable debate as to how SST should be viewed in relation to North Atlantic TC activity^57,58^. For instance^58–60^, showed that hurricane frequency is strongly correlated with the so-called “relative SST”, i.e. the difference between SSTs averaged over the MDR (MDRSST) and global tropical mean SSTs, and that the “relative SST” is a good predictor of North Atlantic TC counts. (2) The El Niño-Southern Oscillation (ENSO) SST anomalies ($^\circ$C) (the El Niño 3 region)^37^, which is governed by large-scale ocean dynamics and coupled ocean-atmosphere interactions from seasonal to interannual variability. ENSO reduces Atlantic TC activity since ENSO induces changes in tropospheric vertical wind shear and the temperature of the upper troposphere^36,38,40,61^. (3) The North Atlantic Multidecadal Oscillation (AMO) sea surface temperature anomalies ($^\circ$C) averaged over the North Atlantic ocean^37^. AMO induces changes in tropical atmospheric circulation which alter the tropospheric vertical shear in the MDR, and positive (warm) and negative (cold) of the AMO can decrease or increase the effect of ENSO on Atlantic TC activity^40,62,63^. This case study also aims at examining how these large-scale climate variables (MDRSST, ENSO, and AMO) are correlated with Atlantic tropical cyclone activity for the interval CE 500–1850. To address this aspect, we use annual paleo-hurricane reconstructions of TC counts (smoothed $\sim 40$ years) from^36^ (Fig. 1). We are aware that there are other climate variables (e.g. the North Atlantic Oscillation—NAO, the stratospheric Quasi-Biennial Oscillation—QBO, the Atlantic Meridional Overturning Circulation—AMOC, among others) that have influence on Atlantic tropical cyclone activity, but we have limited our analysis for the following four reasons: (1) the ultimate goal of this case study is to exemplify the use of a novel statistical and computational tool, the wavelet local multiple correlation (WLMC), to analyse multivariate climate time series; (2) availability of data spanning the time interval in question; (3) paleoclimate reconstructions are complex and it can be problematic (from a technical or methodological point of view) to combine data sets from different studies and archives, and (4) these climate variables have been invoked to explain climate dynamics inferred from paleoclimate records^26^.

## Supplementary information

Supplementary Information.

## Data Availability

In this study we have used previously released, freely available datasets. The datasets were obtained from^36,37^. These are freely available at http://www.meteo.psu.edu/holocene/public_html/Nature09/index.htm and http://www.meteo.psu.edu/holocene/public_html/supplements/MultiproxySpatial09/results/, and in the open repository https://github.com/jomopo/WLMC_climate_time_series. In case of any difficulty in obtaining the datasets mentioned above, the corresponding author can provide the data used upon request.

## Code availability

R codes to produce the figures are available at the open repository https://github.com/jomopo/WLMC_climate_time_series and as Supplementary Information (please look at the Additional information). In case of any difficulty in obtaining the R code mentioned above, the corresponding author can provide the code used upon request.
