# Supplementary material for: Dynamic wavelet correlation analysis for multivariate climate time series
Source: Sci Rep. 2020 Dec 4;10:21277. doi: 10.1038/s41598-020-77767-8 (PMC7718280; doi:10.1038/s41598-020-77767-8)
Supplement: Supplementary file 1 — Supplementary Information. [file 41598_2020_77767_MOESM1_ESM.pdf]

SUPPLEMENTARY MATERIAL to Dynamic wavelet correlation analysis for multivariate climate time series

Josué M. Polanco-Martínez[1*], Javier Fernández-Macho[2], and Martín Medina-Elizalde[3]

[1]Basque Centre for Climate Change (BC3), 48940 Leioa, SPAIN
[2]Dept. of Quantitative Methods, University of the Basque Country, 48015 Bilbao, SPAIN
[3]Dept. of Geosciences, University of Massachusetts - Amherst, MA, USA
[*]josue.polanco@bc3research.org, josue.m.polanco@gmail.com

The `R` **code used to produce the Figures 2–5.** Please use Acrobat Reader or XPDF to copy and paste the code if you want to run it.

```
########################################################################
#:: This script produces the figures related with the wavelet local    #
#:: multiple correlation (WLMC) used in the paper: "Dynamic wavelet    #
#:: correlation analysis for multivariate climate time series" By Josue#
#:: Polanco-Martinez, Javier Fernandez-Macho, & Martin Medina-Elizalde #
#:: Ms. under review in Scientific Reports                             #
#:: Ref: Submission ID 510cee50-a9b3-4dda-b935-0253702278ea            #
########################################################################
#:: Programmed by Josue M. Polanco-Martinez a.k.a jomopo               #
#:: Email: josue.m.polanco@gmail.com                                   #
########################################################################
#:: Copyright (C) 2020 by Josue M. Polanco-Martinez                    #
#:: Some pieces of the code come from J. Fernandez-Macho (2018, 2019)  #
########################################################################
#:: Fernandez-Macho, J. Time-localized wavelet multiple regression and #
#:: correlation. Phys. A: Stat. Mech. its Appl. 492, 1226-1238 (2018), #
#:: https://doi.org/10.1016/j.physa.2017.11.050                        #
#:: Fernandez-Macho, J. Package wavemulcor: Wavelet multiple regression#
#:: and correlation in R. Comput. Sci. & Eng. 21, 63-73 (2019),        #
#:: https://doi.ieeecomputersociety.org/10.1109/MCSE.2018.2875320      #
#:: J. Fernandez-Macho is the author of wavemulcor                     #
#:: Please look at https://CRAN.R-project.org/package=wavemulcor       #
########################################################################
########################################################################
#:: Note for people that don't use R, e.g. users of Python or Matlab:
#:: Users of other languages (e.g. Python or Matlab)  could "call" the
#:: instructions contained in this R code (provided in the Supplementary
#:: Information) within other computational environments, see e.g.:
#:: https://medium.com/@w1ndy/calling-r-libraries-from-python-5ffbf3c3e5a8
#:: https://www.mathworks.com/matlabcentral/fileexchange/5051-matlab-r-link
########################################################################
########################################################################
#:: Please use Acrobat Reader or XPDF to copy and paste the code if you
#:: want to run it!
########################################################################

 rm(list=ls()) # To remove all the objects in the current R session!

########################################################################
# Step 0: install the wavemulcor package version 2.2.1
########################################################################
# wavemulcor version 2.2.1 can be obtained from:
# https://cran.r-project.org/src/contrib/Archive/wavemulcor/
# To install it, please use from R>R CMD INSTALL wavemulcor_2.2.1.tar.gz
# Please note that "wavemulcor" depends on "waveslim"
 library("wavemulcor") # To estimate the WLMC
```

```r
library("plot3D")     # To plot the heat maps

########################################################################
# Step 1: reading the climate time series (source: Mann et al. 2009a,b)
########################################################################
# TC counts (TCStatModelRecon.dat) and MDRSST (mdrrecon.dat) come from:
# http://www.meteo.psu.edu/holocene/public_html/Nature09/index.htm
# NINO3 full (file nino3all) and AMO full (file amoall) come from:
# http://www.meteo.psu.edu/holocene/public_html/supplements/MultiproxySpatial09/results/
# References:
# Mann, M. E., Woodruff, J. D., Donnelly, J. P. & Zhang, Z. Atlantic
# hurricanes and climate over the past 1,500 years. Nature 460,
# 880-883 (2009).
# Mann, M. E. et al. Global signatures and dynamical origins of the
# Little Ice Age and Medieval Climate Anomaly. Science 326, 1256-1260 (2009).
########################################################################

 MDRSST <- read.table("sstmdr_500_1850.txt", header=F)
 sstmdr <- MDRSST[,1:2] #col. 1 and col. 2! Cols. 3 and 4 are the
                        # error/uncertainty of data
 #
 ENSO   <- read.table("nino3all_500_1850.txt", header=F)
 enso   <- ENSO[,1:2]   #col. 1 and col. 2!
 #
 AMO    <- read.table("amoall_500_1850.txt", header=F)
 amo    <- AMO[,1:2]    #col. 1 and col. 2!
 TCount <- read.table("TCStatModelRecon_500_1850.txt", header=F)
 tc     <- TCount[,1:2] # Col. 1 and col. 2

 # Joint data sets (only variables). Col. 1 contains the times/ages
 datin <- cbind(sstmdr[,1], scale(sstmdr[,2]), scale(enso[,2]),
            scale(amo[,2]), scale(tc[,2]))
 N     <- dim(datin)[1] # number of elements of the time series

########################################################################
# Step 2: settings the parameters for the WLMC
########################################################################

 wf     <- "la8"              # wavelet filter or function
 window <- "gaussian"         # window function
 J      <- trunc(log2(N)) - 3 # max. wavelet level
 M      <- trunc(N/8)         # window length N/2^3 (sharper with N/2^4)

 #######################################################################
 # Step 3: Estimating the MODWT, which will be used to estimate the WLMC
 #######################################################################

 xrand1.modwt    <- modwt(datin[,2], wf, J)  # 1: MDRSST
 xrand1.modwt.bw <- brick.wall(xrand1.modwt, wf)

 xrand2.modwt    <- modwt(datin[,3], wf, J)  # 2: ENSO
 xrand2.modwt.bw <- brick.wall(xrand2.modwt, wf)

 xrand3.modwt    <- modwt(datin[,4], wf, J)  # 3: AMO
 xrand3.modwt.bw <- brick.wall(xrand3.modwt, wf)

 xrand4.modwt    <- modwt(datin[,5], wf, J)  # 4: TC
 xrand4.modwt.bw <- brick.wall(xrand4.modwt, wf)

 ########################################################################
 ########################################################################
 # Step 4: jointing the pairs of MODWT for the bi-variate case
 ########################################################################
 ########################################################################

 # Labels and lists
 # 1: SST - ENSO
 lab_output1 <- c("MDRSST_ENSO_2vars")
 xx1         <- list(xrand1.modwt.bw, xrand2.modwt.bw)
 # 2: SST - AMO
```

```
lab_output2 <- c("MDRSST_AMO_2vars")
xx2         <- list(xrand1.modwt.bw, xrand3.modwt.bw)
# 3: SST - TC
lab_output3 <- c("MDRSST_TC_2vars")
xx3         <- list(xrand1.modwt.bw, xrand4.modwt.bw)
# 4: ENSO - AMO
lab_output4 <- c("ENSO_AMO_2vars")
xx4         <- list(xrand2.modwt.bw, xrand3.modwt.bw)
# 5: ENSO - TC
lab_output5 <- c("ENSO_TC_2vars")
xx5         <- list(xrand2.modwt.bw, xrand4.modwt.bw)
# 6: AMO - TC
lab_output6 <- c("AMO_TC_2vars")
xx6         <- list(xrand3.modwt.bw, xrand4.modwt.bw)

# The Big List: jointing all the xx's and labels
XX       <- list(xx1, xx2, xx3, xx4, xx5, xx6)
LAB_OUTP <- list(lab_output1, lab_output2, lab_output3,
                 lab_output4, lab_output5, lab_output6)

# Names of variables by pairs that be used in the plot's titles:
totitle  <- c("MDRSST vs ENSO", "MDRSST vs AMO", "MDRSST vs TC",
              "ENSO vs AMO", "ENSO vs TC", "AMO vs TC")

########################################################################
# Step 5: defining the name of the PDF output file (please note that you
#         will need to create this (plots_to_SciRep) directory (or just
#         remove plots_to_SciRep and save the plots in the current directory),
#         it's also defined the setting for the plot output.
########################################################################

if(1){ # To activate (if(1)) this piece of code (if(0) deactivate)

# Please look at Figures 2 & 4 (bi-variate case)
pdf(file=paste("./plots_to_SciRep/wlmc_2vars_500_1850.pdf", sep=""),
    width=12, height=9)
par(oma=c(0, 0, 0, 1), mar=c(4, 6.5, 2, 0.5) + 0.1)

########################################################################
# Step 6: "the action", the next pieces of code are used to estimate the
#          WLMC and to plot the WLMC's outputs as heat maps.
########################################################################

# This loop is used to estimate the WLMC and to plot theirs heat maps
# for the six pairs: 1) "MDRSST vs ENSO", 2) "SSTDMR vs AMO",
# 3) "MDRSST vs TC", 4) "ENSO vs AMO", 5) "ENSO vs TC", and 6) "AMO vs TC"
for (i in 1:6) {

########################################################################
# The function "wave.local.multiple.correlation" from the "wavemulcor"
# package is used to estimate the WLMC
########################################################################
xy.mulcor <- wave.local.multiple.correlation(XX[[i]], M, window=window,
              ymaxr=NULL)
val       <- as.matrix(xy.mulcor$val)   # correlation coefficients
lo        <- as.matrix(xy.mulcor$lo)    # CI lower bounds
up        <- as.matrix(xy.mulcor$up)    # CI upper bounds
YmaxR     <- as.matrix(xy.mulcor$YmaxR) # The index numbers of the
#                        variable whose correlation is calculated
#                        against a linear combination of the rest
########################################################################

# Parameters to be used to plot the heat maps. Please note that
# scale.names are defined only for 10 levels (wavelet scales)!
scale.names <- c("[2-4]", "(4-8]", "(8-16]", "(16-32]", "(32-64]",
                 "(64-128]", "(128-256]", "(256-512]", "(512-1024]",
                 "(1024-2048]")
scale.names <- c(scale.names[1:J],"Smooth")
xlab        <- "Years (CE)"
ylab        <- ""
```

```
 ######################################################################
 # This piece of code is used to put blank marks to indicate that these
 # points (correlation coefficients) are not statistically significant
 # (outside of the 95% confidence interval)
 for (l in 1:J) {
  id.0 <- which(lo[,l] <= 0 & up[,l] >= 0)
  val[id.0,l] <- NA
 }
 ######################################################################
 # This piece of code is used to plot the WLMC heat maps

 image2D(z=val, x=datin[,1], y=1:ncol(val), cex=1.45, cex.lab=1.5,
  main=totitle[i], colkey=list(cex.axis=1.35), xlab=xlab, ylab=ylab,
  axes=FALSE, rasterImage=FALSE, contour=list(lwd=2, col=1))
 axis(side=1, at=pretty(datin[,1]), cex.axis=1.35)
 axis(side=2, at=1:ncol(val),labels=scale.names, las=1, cex.axis=1.35)
 mtext(2, text="Periods (Years)", line=5.25, cex=1.5)

 } # End of "for"

 dev.off() # Closing the PDF device (to save the PDF file)
} # End of if(1 or 0)

 ##########################################################################
 ##########################################################################
 # Step 7: jointing the triads of MODWT for the three-variate case
 ##########################################################################
 ##########################################################################

 # Labels and lists
 # 1: SST - ENSO -  AMO
 lab_output1 <- c("MDRSST_ENSO_AMO_3vars")
 xx1         <- list(xrand1.modwt.bw, xrand2.modwt.bw, xrand3.modwt.bw)
 # 2: SST - ENSO - TC
 lab_output2 <- c("MDRSST_ENSO_TC_3vars")
 xx2         <- list(xrand1.modwt.bw, xrand2.modwt.bw, xrand4.modwt.bw)
 # 3: SST - AMO - TC
 lab_output3 <- c("MDRSST_AMO_TC_3vars")
 xx3         <- list(xrand1.modwt.bw, xrand3.modwt.bw, xrand4.modwt.bw)
 # 4: ENSO - AMO - TC
 lab_output4 <- c("ENSO_AMO_TC_3vars")
 xx4         <- list(xrand2.modwt.bw, xrand3.modwt.bw, xrand4.modwt.bw)

 # The Big List: jointing all the xx's and labels
 XX       <- list(xx1, xx2, xx3, xx4)
 LAB_OUTP <- list(lab_output1, lab_output2, lab_output3, lab_output4)

 # Names of variables of the triads that be used in the plot's titles
 totitle  <- c("MDRSST, ENSO, AMO", "TC <- MDRSST, ENSO",
              "TC <- MDRSST, AMO", "TC <- ENSO, AMO")

 ##########################################################################
 # Step 8: defining the name of the PDF output file (please note that you
 #         will need to create this (plots_to_SciRep & 2vars) directory,
 #         it's also defined the settings for the plot output.
 ##########################################################################

if(1){ # To activate (if(1)) this piece of code (if(0) deactivate)

 # Please look at Figure 3 and 5
 pdf(file=paste("./plots_to_SciRep/wlmc_3vars_500_1850.pdf", sep=""),
     width=12, height=9)
 par(oma=c(0, 0, 0, 1), mar=c(4, 6.5, 2, 0.5) + 0.1)

 ##########################################################################
 # Step 9: "the action", the next pieces of code are used to estimate the
 #         WLMC and to plot the WLMC's outputs as heat maps.
 ##########################################################################
```

```
# We first estimate the WLMC for the triad: "MDRSST, ENSO, AMO"
# (Figure 3) and the other 3 triads (Figure 5)

# This loop is used to estimate the WLMC and to plot theirs heat maps
# for the four triads: 1) "MDRSST, ENSO, AMO", 2) "TC <- MDRSST, ENSO",
# 3) "TC <- MDRSST, AMO", and 4) "TC <- ENSO, AMO"
for (i in 1:4) {

########################################################################
# The function "wave.local.multiple.correlation" from the "wavemulcor"
# package is used to estimate the WLMC
########################################################################
if(i == 1)  # XX[[1]] contain the MODWT of MDRSST, ENSO and AMO
xy.mulcor <- wave.local.multiple.correlation(XX[[i]], M, window=window,
              ymaxr=NULL) # This option is used since we have not defined
                          # a priori specific climate variable that would
                          # maximize the multiple correlation
if(i != 1) # XX[[2,3,4]] are the other triads
xy.mulcor <- wave.local.multiple.correlation(XX[[i]], M, window=window,
              ymaxr=3)    # We want to maximize the multiple correlation
                          # with respect to TC (dependent variable)
val       <- as.matrix(xy.mulcor$val)   # correlation coefficients
lo        <- as.matrix(xy.mulcor$lo)    # CI lower bounds
up        <- as.matrix(xy.mulcor$up)    # CI upper bounds
YmaxR     <- as.matrix(xy.mulcor$YmaxR) # The index numbers of the
#                         variable whose correlation is calculated
#                         against a linear combination of the rest
########################################################################

# Parameters to be used to plot the heat maps. Please note that
# scale.names are defined only for 10 levels (wavelet scales)!
scale.names <- c("[2-4]", "(4-8]", "(8-16]", "(16-32]", "(32-64]",
                 "(64-128]", "(128-256]", "(256-512]", "(512-1024]",
                 "(1024-2048]")
scale.names <- c(scale.names[1:J],"Smooth")
xlab        <- "Years (CE)"
ylab        <- ""

####################################################################
# This piece of code is used to put blank marks to indicate that these
# points (correlation coefficients) are not statistically significant
# (outside of the 95% confidence interval)
for (l in 1:J) {
 id.0 <- which(lo[,l] <= 0 & up[,l] >= 0)
 val[id.0,l] <- NA
}
####################################################################
# This piece of code is used to plot the WLMC heat maps (Fig. 3, left)

image2D(z=val, x=datin[,1], y=1:ncol(val), cex=1.45, cex.lab=1.5,
 main=totitle[i], colkey=list(cex.axis=1.35), xlab=xlab, ylab=ylab,
 axes=FALSE, rasterImage=FALSE, contour=list(lwd=2, col=1))
axis(side=1, at=pretty(datin[,1]), cex.axis=1.35)
axis(side=2, at=1:ncol(val),labels=scale.names, las=1, cex.axis=1.35)
mtext(2, text="Periods (Years)", line=5.25, cex=1.5)

####################################################################
# This is piece of code is used ONLY to generate Fig. 3 (right)!
if(i == 1) {
par(mfrow=c(1,1))
par(oma=c(0, 0, 0, 1), mar=c(4, 6.5, 2, 2.75) + 0.1)

rang_colb <- sort(unique(unlist(apply(YmaxR, 2, unique))))
Nragcb    <- length(rang_colb)
at_lab    <- 1:3
LAB_NAM   <- c("MDRSST", "ENSO", "AMO")
rang_colb <- c("blue", "green", "red")

image2D(z=YmaxR, x=datin[,1], y=1:ncol(val), cex=1.45, cex.lab=1.5,
 main=totitle[1], xlab=xlab, ylab=ylab, axes=FALSE, rasterImage=FALSE,
```

```
  col=rang_colb, colkey=list(at=at_lab, labels=(LAB_NAM), cex.axis=1.35))
 axis(side=1, at=pretty(datin[,1]), cex.axis=1.35)
 axis(side=2, at=1:ncol(val),labels=scale.names[1:8], las=1, cex.axis=1.35)
 mtext(2, text="Periods (Years)", line=5.25, cex=1.5)
 }
 ######################################################################

 } # End of "for"

 dev.off() # Closing the PDF device (to save the PDF file)
} # End of if(1 or 0)

 ##########################################################################
 ##########################################################################
 # Step 9: jointing the tetra-pairs of MODWT for the tetra-variate case
 ##########################################################################
 ##########################################################################

 # Labels and lists
 # 1: SST - ENSO - AMO - NAO
 lab_output1 <- c("SSTMDR_ENSO_AMO_TC_4vars")
 xx1         <- list(xrand1.modwt.bw, xrand2.modwt.bw, xrand3.modwt.bw,
                     xrand4.modwt.bw)

 # The Big List
 XX       <- list(xx1)
 LAB_OUTP <- list(lab_output1)
 # Names of variables for the tetra-variate case:
 totitle  <- c("TC <- SSTMDR, ENSO, AMO")

 ##########################################################################
 # Step 10: defining the name of the PDF output file (please note that you
 #           will need to create this (plots_to_SciRep) directory,
 #           it's also defined the setting for the plot output.
 ##########################################################################

if(1){ # To activate (if(1)) this piece of code (if(0) deactivate)

 # Please look at Figure 5 (right and below)
 pdf(file=paste("./plots_to_SciRep/wlmc_4vars_500_1850.pdf", sep=""),
     width=12, height=9)
 par(oma=c(0, 0, 0, 1), mar=c(4, 6.5, 2, 0.5) + 0.1)

 ##########################################################################
 # Step 11: "the action", the next pieces of code are used to estimate the
 #           WLMC and to plot the WLMC's outputs as heat maps.
 ##########################################################################

 # We estimate the WLMC for the variables: "MDRSST, ENSO, AMO, and TC"

 ##########################################################################
 # The function "wave.local.multiple.correlation" from the "wavemulcor"
 # package is used to estimate the WLMC
 ##########################################################################
 xy.mulcor <- wave.local.multiple.correlation(XX[[1]], M, window=window,
              ymaxr=4)     # We want to maximize the multiple correlation
                           # with respect to TC (dependent variable)
 val       <- as.matrix(xy.mulcor$val)    # correlation coefficients
 lo        <- as.matrix(xy.mulcor$lo)     # CI lower bounds
 up        <- as.matrix(xy.mulcor$up)     # CI upper bounds
 YmaxR     <- as.matrix(xy.mulcor$YmaxR) # The index numbers of the
 #                         variable whose correlation is calculated
 #                         against a linear combination of the rest
 ##########################################################################

 # Parameters to be used to plot the heat maps. Please note that
 # scale.names are defined only for 10 levels (wavelet scales)!
 scale.names <- c("[2-4]", "(4-8]", "(8-16]", "(16-32]", "(32-64]",
                 "(64-128]", "(128-256]", "(256-512]", "(512-1024]",
                 "(1024-2048]")
```

```
 scale.names <- c(scale.names[1:J],"Smooth")
 xlab        <- "Years (CE)"
 ylab        <- ""

 ####################################################################
 # This piece of code is used to put blank marks to indicate that these
 # points (correlation coefficients) are not statistically significant
 # (outside of the 95% confidence interval)
 for (l in 1:J) {
  id.0 <- which(lo[,l] <= 0 & up[,l] >= 0)
  val[id.0,l] <- NA
 }
 ####################################################################
 # This piece of code is used to plot the WLMC heat maps

 image2D(z=val, x=datin[,1], y=1:ncol(val), cex=1.45, cex.lab=1.5,
  main=totitle, colkey=list(cex.axis=1.35), xlab=xlab, ylab=ylab,
  axes=FALSE, rasterImage=FALSE, contour=list(lwd=2, col=1))
 axis(side=1, at=pretty(datin[,1]), cex.axis=1.35)
 axis(side=2, at=1:ncol(val),labels=scale.names, las=1, cex.axis=1.35)
 mtext(2, text="Periods (Years)", line=5.25, cex=1.5)

 dev.off() # Closing the PDF device (to save the PDF file)
} # End of if(1 or 0)
```
